# Investigating the Effect of Aging on the Viscosity of Tendon Fascicles and Fibers

**DOI:** 10.3389/fbioe.2019.00107

**Published:** 2019-05-15

**Authors:** Nikolaos Karathanasopoulos, Jean-francois Ganghoffer

**Affiliations:** ^1^Chair of Computatonal Modeling of Materials in Manufacturing, ETH Zurich, Zurich, Switzerland; ^2^LEM3, CNRS, Université de Lorraine, Metz, France

**Keywords:** tendon, relaxation, viscosity, aging, fascicle, fiber, matrix

## Abstract

In the current work, we investigate the effect of aging on the viscosity of tendon subunits. To that scope, we make use of experimental relaxation curves of healthy and aged tendon fascicles and fibers, upon which we identify the viscosity parameters characterizing the time-dependent behavior of each tendon subunit. We subsequently combine the obtained results with analytical viscoelastic homogenization analysis methods to extract information on the effective viscous contribution of the embedding matrix substance at the fiber scale. The results suggest that the matrix substance plays a significant role in the relaxation process of the upper tendon subunits both for aged and healthy specimens. What is more, the viscosity coefficients computed for the fibrillar components indicate that aging leads to a viscosity reduction that is statistically significant for both fascicles and fibers. Its impact is more prominent for the lower hierarchical scale of fibers. As such, the reduced stress relaxation capability at the tendon macroscale is to be primarily attributed to the modified viscosity of its inner fibrillar subunits rather than to the matrix substance.

## Introduction

**Figure 1:**
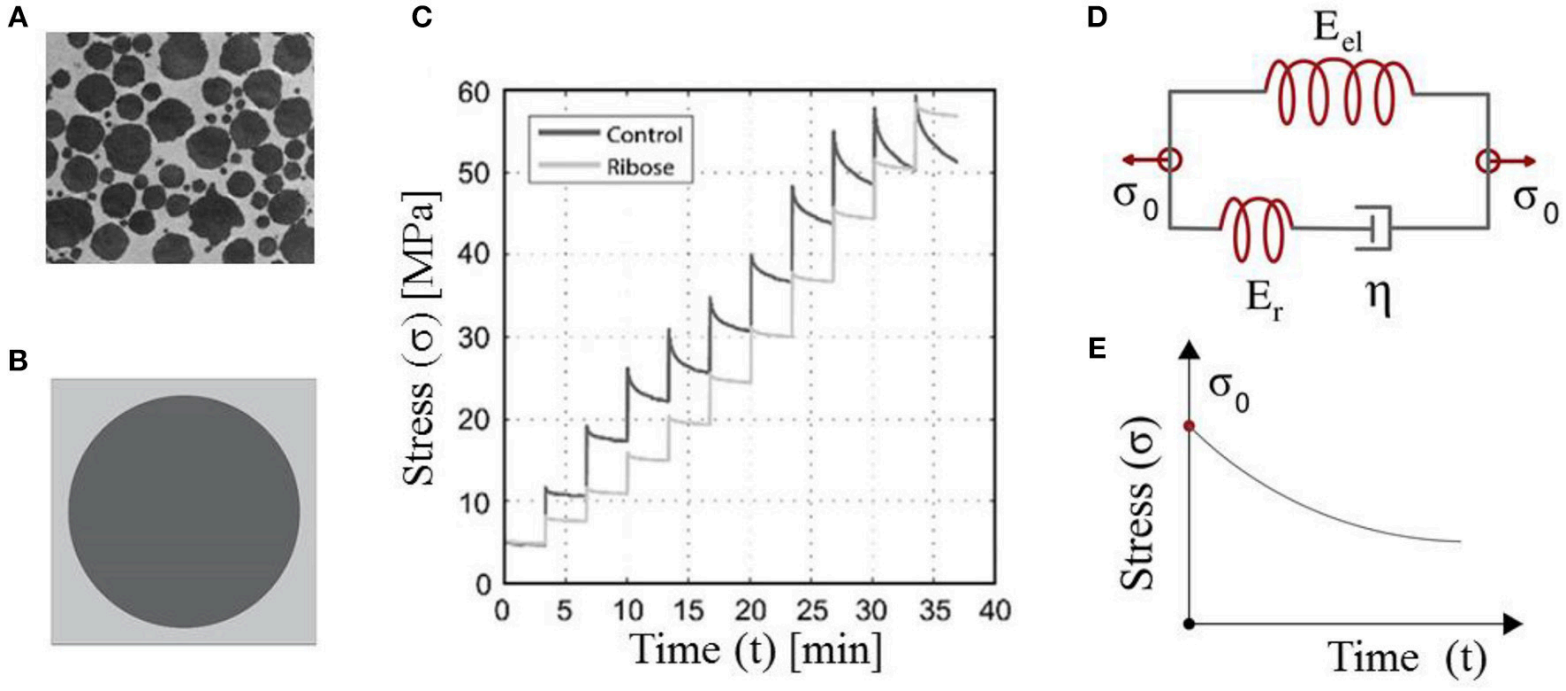
Tendon fibers immersed in a matrix substance **(A)**. Maceri et al. (2012) and Gautieri et al. (2017) defining a certain averaged, unit-cell fiber content (Karathanasopoulos et al., 2019) **(B)**. The short-time relaxation response of *in-vitro* human tendon fascicles upon repetitive incremental loading steps **(C)**. Gautieri et al. (2017) and their theoretical representation by means of Maxwell-type relaxing elements with a primal material viscosity η (Lakes, 2009) **(D,E)**. **(A,C)** are reproduced with the permission of the copyright holder [RightsLink/Elsevier].

The multiscale structure of tendons plays a functional role in the transfer of forces from the muscles to the bones (Maceri et al., 2012; Ge et al., 2018). The fascicles contained within the tendon unit are composed of fibers immersed in a matrix substance (Figure 1A) (Goh et al., 2008). Disease or age-related changes taking place at the tendon's molecular scale are expressed in terms of modified mechanical properties at the upper tendon scales, namely at the fiber and fascicle scale (Bailey, 2001; Zhang et al., 2014).

In order to characterize the tendon's mechanical attributes, tensile experiments have been carried out at different inner tendon hierarchies. In particular, quasi-static stress-strain curves have been used to provide estimates for the elastic modulus of fascicles and fibers (Kato et al., 1989; Gentleman et al., 2004; Lavagnino et al., 2005; Svensson et al., 2010). However, the linear elastic stress-strain response does not suffice to describe the substantial stress relaxation capabilities observed for tendons (Salathe and Arangio, 1990). Viscoelasticity has provided the theoretical basis for the characterization of the tendon's relaxation behavior (Elliott et al., 2003; Machiraju et al., 2006; Screen, 2008; Shen et al., 2011), mathematically described as a function of both elastic and viscous parameters (Taylor et al., 1970; Christensen, 1982). The tendon's viscoelastic properties have been shown to differ among its lower and upper scales, with the embedding matrix substance to play a significant role in the multiscale effective relaxation behavior (Karathanasopoulos et al., 2019).

It is up to now well-established that the aging process, as well as deceases such as diabetes, result in functional changes, which have been directly related to increased tendon damage and injury (Dressler et al., 2002), as well as to reduced healing capacity (Bedi et al., 2010). Such phenomenological effects arise from inner structural changes, which alter the mechanical properties of the tendons's inner scales (Snedeker and Foolen, 2017). In particular, it has been shown that aging induces non-enzymatic cross-linkings upon a glycation process which modifies the loading capacity and time-dependent attributes at the tropocollagen scale (Gautieri et al., 2013; Vesentini et al., 2013; Nair et al., 2014), thus already at the innermost structural building block of tendons. What is more, it has been experimentally demonstrated that aging primarily affects the viscoelastic properties and the failure mode (Li et al., 2013) of tendon fibrils, fibers and fascicles (Gautieri et al., 2017), an observation made for both human and animal tissues (Hansen et al., 2010; Maceri et al., 2012). Contrariwise, the elastic modulus of aged tendons remains practically unaffected for all of the previously reported subunits (Legerlotz et al., 2013; Fessel et al., 2014).

While the qualitative effects of aging on tendons have been long identified and relevant stress measurements are available, quantitative estimates of the resulting modifications in the effective viscosities of the different inner tendon scales remain to be provided. Information of the kind is of primal importance, not only for the understanding of the mechanical behavior of the tendon's inner fibrillar components (Karathanasopoulos and Ganghoffer, 2019a), but also for the embedding matrix substance. The latter has been shown to play a primal role both in the relaxation process (Karathanasopoulos et al., 2019) and in the progression of tissue related deceases (Snedeker and Gautieri, 2014). However, mechanical testing at the embedding matrix substance scale is rather infeasible (Ault et al., 1992), so that no direct experimental data are available. As a result, its mechanical properties are estimated either though physics-motivated analytical models (Ault et al., 1992) or multiscale modeling inference techniques (Karathanasopoulos and Hadjidoukas, 2019b; Karathanasopoulos et al., 2019).

In the current work, we make use of experimental data which we combine with viscoelastic mechanical models to provide quantitative estimates for the effective viscosity of healthy and aged tendon subunits. In particular, in section Methodology, we summarize the theoretical framework to compute the relaxation behavior of tendon subunits. Thereupon, we compute the viscoelastic parameters that characterize the relaxation behavior of healthy and aged tendon fascicles and fibers, quantifying the relevant experimental uncertainty (see sections Relaxing Healthy and Aging Fascicles and Fiber Scale Aging Relaxation) and the statistical significance of the observed alterations (see Statistical Significance of the Effective Viscosity Alterations Upon Aging). Combing the mechanical data with analytical, homogenization analysis techniques, we furnish estimates for the effective viscosity of the embedding matrix substance at the fiber scale both for the healthy (control) and for the aged tendon specimens (see section Effective Viscosity Contributions of the Embedding Matrix). In section Discussion, we comment on the obtained results, providing considerable insights in the effect of aging at the different tendon inner scales and conclude in section Conclusions.

## Methodology

The fibrous, matrix-embeded structure of fascicles and fibers (Figure 1A) (Ge et al., 2018) has allowed for the tendon subunits to be characterized as naturally architected, two-phase composite materials (Maceri et al., 2012). The fibrillar components are in a certain relative density to the embedding matrix substance, so that a unit-cell with a fibrous fraction *f*_*r*_ can be defined (Figure 1B) (Ganghoffer et al., 2016; Karathanasopoulos et al., 2017, 2019).

Each material phase can be considered to be in the general case of viscoelastic nature, with elastic and viscous properties *E*_*f*_, *E*_*m*_ and η_*f*_, η_*m*_ for the fibrillar and matrix components accordingly. As such, the effective homogenized viscoelastic response of the matrix embedded tendon fibers is characterized by the following constitutive equation (Ganghoffer et al., 2016; Karathanasopoulos et al., 2019):

(1) $$\begin{array}{l} \bar{\sigma }=\langle E\rangle \bar{\varepsilon }+\langle \eta \rangle \dot{\bar{\varepsilon }}+\langle \eta \rangle \frac{E_{f}E_{m}}{\eta_{f}\eta_{m}}\left(\frac{\langle \eta /E^{2}\rangle }{\langle 1/E\rangle }-\frac{\langle 1/E\rangle }{\langle 1/\eta \rangle }\right)\\ \;\int_{0}^{t}exp\left(-\frac{E_{f}}{\eta_{f}}\frac{E_{m}}{\eta_{m}}\frac{\langle \eta \rangle }{\langle E\rangle }\left(t-s\right)\right)\dot{\bar{\varepsilon }}(s)ds \end{array}$$

Where in Equation (1), components with a bar stand for the homogenized strain and stress $\bar{\varepsilon }$ and $\bar{\sigma }$, while brackets for the homogenized elastic and viscous moduli, 〈*E*〉 and 〈η〉 accordingly. The latter depend on the volumetric fraction of the two phases, as follows (Ganghoffer et al., 2016):

(2) $$\langle \eta \rangle =\eta_{fasc}=\eta_{f}f_{r}+\eta_{m}\left(1-f_{r}\right),\langle E\rangle =E_{fasc}=E_{f}f_{r}+E_{m}\left(1-f_{r}\right)$$

Given the homogenized elastic and viscous material parameters, the time-dependent response of the tendon subunits is characterized by Maxwell-type relaxation kernels (Figures 1D,E) (Christensen, 1982; Lakes, 2009). The primal relaxation behavior of the viscoelastic structure is a kernel function of its elastic and viscous parameters, defined as follows (Christensen, 1982; Lakes, 2009):

(3) $$E\left(t\right)=E_{el}+E_{r}e^{-\frac{E_{r}}{\eta }t},E\left(t=0\right)=E_{el}+E_{r}$$

where in Equation (3), *E*_*r*_ stands for the relaxing modulus part, which is equal to the substraction of the elastic modulus part *E*_*el*_ (the modulus part remaining at the end of the relaxation experiment) from the initial modulus *E*_*r*_ = *E*(*t* = 0) − *E*_*el*_ (Figure 1). For *t* = 0, the elastic modulus is equal to its initial homogenized, non time-dependent value, as indicated by Equation (3). Equation (3) applies to both fascicles (fasc) and fibers (f), with the viscosity parameter to be denoted as η_*fasc*_ and η_*f*_ accordingly.

While the initial modulus can be directly retrieved out of experimental testing, information on the viscous modulus η of Equation (3) needs to be extracted making use of the relaxation curves (Figure 1C). For the viscous modulus to be identified, the viscoelastic parameter η in the time-dependent modulus evolution of Equation (3) is optimized to meet the experimental modulus evolution Ê(*t*) of each relaxation curve (Figure 3C, Li et al., 2013; Gautieri et al., 2017) using a control timestep of ten seconds between the starting *t*_*s*_ and final time *t*_*f*_ of each relaxation experiment, upon the following loss function:

(4) $$\arg\;\min L=\sum_{t_{s}}^{t_{f}}\left\|E\left(t\right)-\hat{E}(t)\right\|$$

For the minimization of Equation (4), a standart derivative-free method has been employed using Matlab 2018a. In the Sections to follow, we make use of the definitions of Equations (1)–(4) along with experimental data provided in Li et al. (2013) and Gautieri et al. (2017) to compute the primal viscosity coefficient of tendon fascicles (see section Relaxing Healthy and Aging Fascicles) and fibers (see section Fiber Scale Aging Relaxation), as well as to extract information on the effective viscous contribution of the embedding matrix substance (see section Effective Viscosity Contributions of the Embedding Matrix).

In order to assess the significance of the identified viscosity changes among the control and aged tendon fascicles and fibers, we carry out a Welch's *t*-test in section Statistical Significance of the Effective Viscosity Alterations Upon Aging. The main parameters of the *t*-test, namely the t value and the associated number of degrees of freedom ν are given as follows (Fagerland, 2012):

(5) $$\begin{array}{l} t=\left(\mu_{c}-\mu_{a}\right)/\sqrt{\sigma_{c}^{2}/N_{c}+\sigma_{a}^{2}/N_{a}}\\ \nu ={\left(\sigma_{c}^{2}/N_{c}+\sigma_{a}^{2}/N_{a}\right)}^{2}/(\sigma_{c}^{4}/\left(N_{c}^{2}\left(N_{c}-1\right)\right)\\ \;+\;\sigma_{a}^{4}/\left(N_{a}^{2}\left(N_{a}-1\right)\right)) \end{array}$$

where μ_*c*_ and μ_*a*_ in Equation (5) stand for the mean value (μ) of a certain control (c) and aged (a) quantity, while σ and *N* stand for the standard deviation and the sample size accordingly.

## Results

### Relaxing Healthy and Aging Fascicles

**Figure 2:**
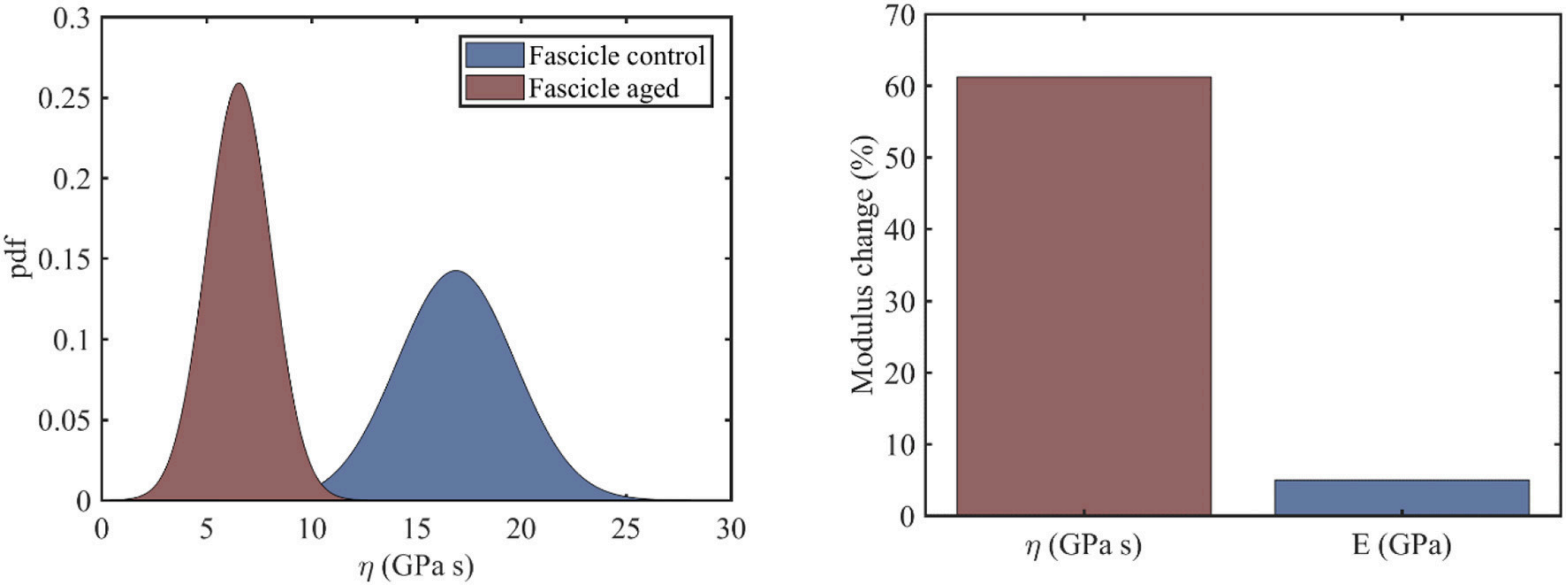
Probability density function of the viscosity coefficients of healthy and aged tendon fascicles (left) and the percentage reduction of its mean value (right).

In the current section, we identify the viscosity coefficient η of relaxing healthy and aged fascicles, using Equation (3) along with the experimental data provided in Gautieri et al. (2017) and depicted in Figure 1C. In particular, we compute the viscosity coefficient η for each of the healthy and ribosed fascicle relaxation curves, carrieη d out within the linear elastic strain range as low as 1.8% and up to 4.8%, upon strain increments of 0.6% (relaxation experiments in between 10 and 30 min in Figure 1C). Each relaxation experiment has been conducted for a total duration of 200 s, so that the total number N of relaxation experiments for which the viscosity value is identified is *N* = 6. The mean elastic modulus (*t* = 0) of the fascicles has been computed to be 〈*E*〉_*c*_ = 892*MPa* and 〈*E*〉_*a*_ = 942*MPa* for control (c) and aged (a) fascicles, accordingly (Li et al., 2013; Gautieri et al., 2017). In Figure 2 (left), we provide the computed mean and standard deviation of the probability density function (pdf) for the coefficient η of the healthy and aged fascicles using Equation (4). In Figure 2 (right), we provide the percentage reduction of the mean viscosity value η, compared to the percentage change of the elastic modulus E.

The left subplot of Figure 2 suggests a clear separation of the probability distributions of the viscosity coefficients for the healthy and the aged fascicles. More specifically, for the control healthy fascicle, a mean viscous coefficient of $\eta_{fasc}^{c}=17GPas$ has been identified with a standard deviations of 2.8 *GPas*. Accordingly, for aged fascicles, a mean effective viscosity coefficient of $\eta_{fasc}^{a}=6.5GPas$ along with a standard deviation of 1.6 *GPas* has been obtained. The percentage difference for the mean value of the fascicle viscoelastic modulus η_*fasc*_ between the healthy and aged tendon tissue is in the order of 60% (Figure 2, right), contrary to the rather negligible variation of 5% for its elastic modulus 〈*E*〉. The relaxation curves for aged and healthy fascicles arising from the identified fascicle viscosity η_*fasc*_ are provided for completeness in Appendix A.

### Fiber Scale Aging Relaxation

**Figure 3:**
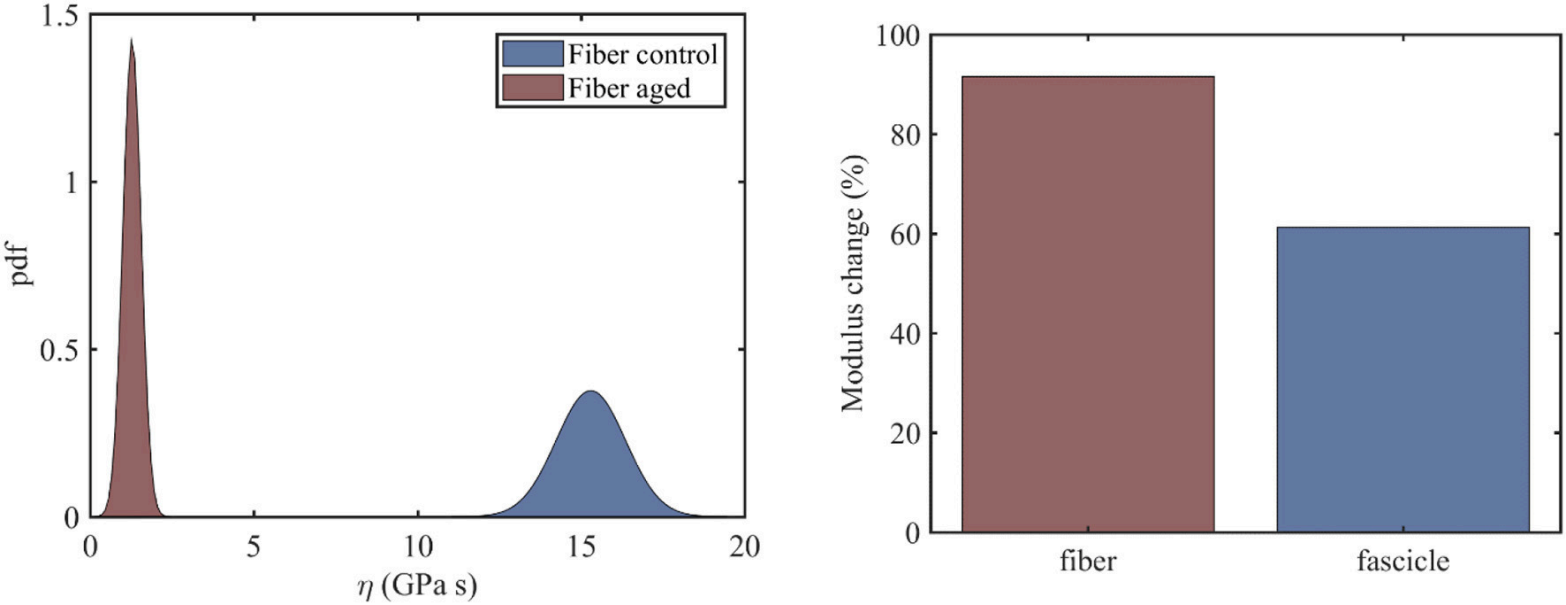
Probability distribution of the viscosity coefficients η of healthy and aged tendon fibers (left) along with the percentage change upon aging of the mean viscous moduli value for fibers and fascicles (right).

At the fiber scale, we identify the viscosity coefficient η_*f*_ using Equation (3), so that Equation (3) simplifies to $E_{f}\left(t\right)=E_{el}+E_{r}e^{-\left(\frac{E_{r}}{\eta_{f}}\right)*t}$. The fiber scale relaxation curves for healthy and aged tendon fibers are provided in Li et al. (2013). For the fiber viscosity computations, a total of six (*N* = 6) relaxation experiments within the linear elastic strain range is used, in particular for strain magnitudes in between 2 and 4.5%, upon strain increments of 0.5%. In the left subplot of Figure 3, we provide the computed probability density functions for the viscosity values η of healthy and aged tendon fiber specimens, while in Figure 3 (right) the percentage reduction in the mean viscosity values among healthy and aged tendon fiber and fascicle (see section Relaxing Healthy and Aging Fascicles) specimens.

**Figure 4:**
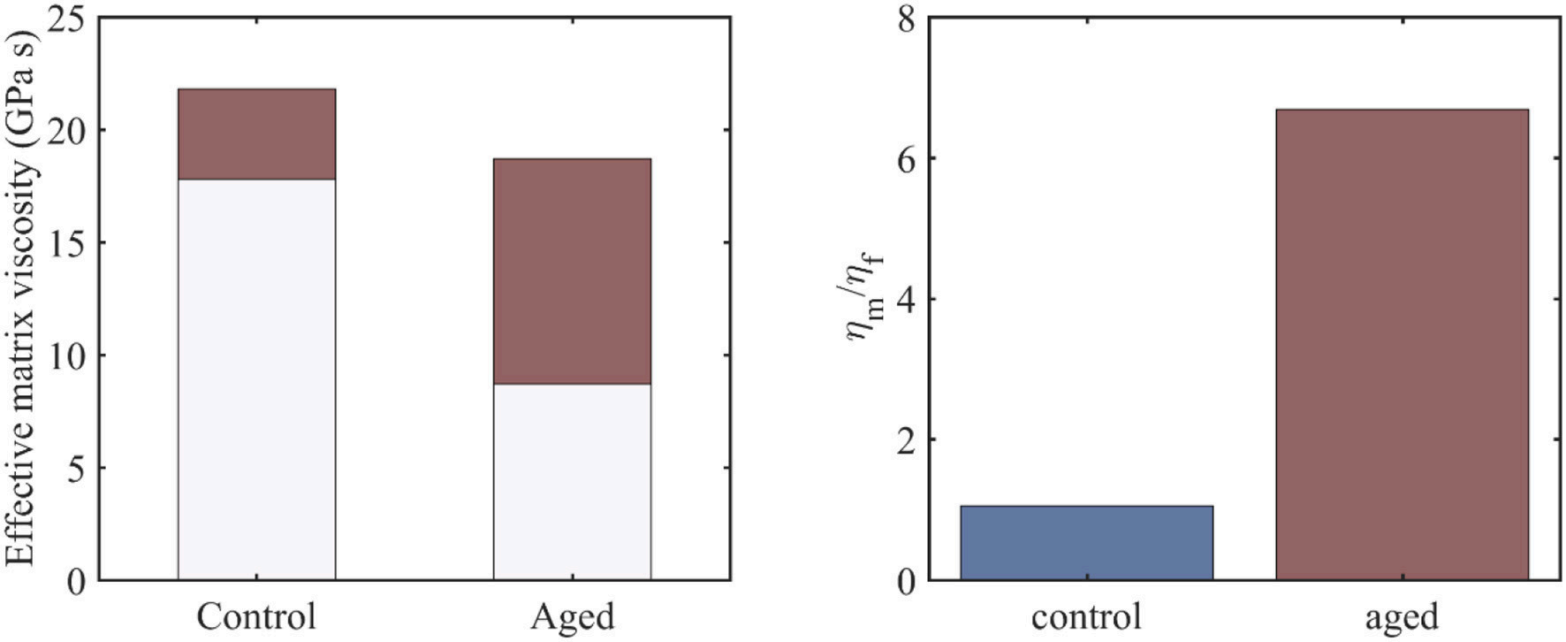
The range of the effective viscosity of the embedding matrix –depicted in red- for control and aged tendon fascicle specimens and an *f*_*r*_ value in between 0.3 and 0.7 (left), along with the ratio of the fiber to the embedding matrix viscosities for a fiber fraction of *f*_*r*_ = 0.3 (right).

Figure 3 (left) indicates a complete separation of the viscoelastic coefficients pertaining to healthy and aged fiber specimens. In particular, while for the control fibers, a mean viscous coefficient value of $\eta_{f}^{c}=15.3GPas$ is computed, for the aged ones, a value of $\eta_{f}^{a}=1.3GPas$ is obtained. What is more, the standard deviation of the viscosity of the aged fibers is considerably smaller (0.276*GPas*) than the one obtained for the control tendon fibers (1.05*GPas*). The mean viscosity coefficient for aged fiber specimens is considerably smaller than the corresponding η value at the fascicle scale (Figure 3, left). Moreover, the percentage reduction of the viscoelastic parameter upon aging is considerably higher at the fiber scale compared to the fascicle scale (Figure 4, right). The computed fiber-scale relaxation curves are provided for completeness in Appendix A.

### Effective Viscosity Contributions of the Embedding Matrix

For the viscous contribution of the embedding matrix substance to be quantified, we make use of the previously obtained viscosity results at the fiber and fascicle scale (Figures 2, 3), which we couple with the analytical homogenization based predictions of Equations (1) and (2). In particular, we use the mean values of the computed viscosity coefficients η_*fasc*_ = 〈η〉 at the fascicle scale (Figure 2) to infer the viscous contribution of the embedding matrix at the fiber scale η_*m*_, using the results of Figures 2, 3 and Equation (2). For the computations, we allow for the fiber content entering *f*_*r*_ Equation (2) to vary in between 0.3 and 0.7 (Maceri et al., 2012; Li et al., 2013). In Figure 4 (left), we depict the range of values –in red- calculated for the effective viscosity of the embedding matrix η_*m*_ for control and aged specimens, while in Figure 4 (right), the ratio of the viscosity of the matrix to the one of the fiber (η_*m*_/η_*f*_) for a fibrillar fraction of *f*_*r*_ = 0.3.

Figure 4 provides an estimate of the effective viscosity of the embedding matrix η_*m*_ at the tendon fiber scale, both for the control and aged tendons. For the former, a matrix viscosity value between 18 and 22 *GPas* is obtained for low and high fiber content values accordingly (Figure 4, left). For their aged counterparts, a considerably wider range of values is computed, as low as 8.5 an up to 18.5 *GPas* (Figure 4, left). For a given fiber content value *f*_*r*_ (e.g., for *f*_*r*_ = 0.3, corresponding to the minimum value of the bars in Figure 4, left), the effective viscosity of the embedding matrix of aged specimens is considerably lower than the one of the control ones (Figure 4, left). However, for the aged tendons, the relative viscous embedding matrix contribution is considerably higher than the one of control fascicle specimens, as the ratio of the effective matrix viscosity to the fiber matrix viscosity η_*m*_/η_*f*_ in Figure 4 (right) suggests.

### Statistical Significance of the Effective Viscosity Alterations Upon Aging

We subsequently assess the significance of the computed viscosity changes among the control and aged tendon fascicles and fibers, using the *t*-test metrics, summarized in Equation (5). At the fascicle scale, we compute a t value that is *t* = 7.98, along with an ν value of ν = 7.95, using the mean viscosity η_*fasc*_ and standard deviation values provided in section Relaxing Healthy and Aging Fascicles. The values relate to a *p*-value that is lower than 0.01 within a 5% significance interval, suggesting a significant viscosity difference between the control and aged fascicle groups. Performing the same analysis for the viscosity parameters computed at the fiber scale (see section Fiber Scale Aging Relaxation), we obtain a t value that is *t* = 31.5 along with an ν value of ν = 5.7. The values pair to a p-value that is lower than 10^−4^ within a 5% significance interval, indicating a highly significant difference between the control and aged tendon fiber groups. The results are summarized in Table 1.

**Table 1:** Statistical significance of the alterations in the viscosity parameters computed for control and aged tendon fascicles (see section Relaxing Healthy and Aging Fascicles) and fibers (see section Fiber Scale Aging Relaxation) using t-test statistics.

**Scale**	***t*-value**	**ν value**	***p*-value**
Fascicle	7.98	7.95	<0.01
Fiber	31.5	5.7	<0.0001

## Discussion

The results of sections Relaxing Healthy and Aging Fascicles, Fiber Scale Aging Relaxation, and Effective Viscosity Contributions of the Embedding Matrix provide experimentally-based, quantitative estimates of the effect of aging on the time-dependent, viscous properties of tendon subunits. In particular, the viscosities at the scale of fascicles (see section Relaxing Healthy and Aging Fascicles) and fibers (see section Fiber Scale Aging Relaxation), as well as the effective viscoelastic contribution of the fiber embedding matrix substance (see section Effective Viscosity Contributions of the Embedding Matrix) are assessed.

The experimental data at the fascicle scale (Figure 1C) yield a mean viscosity value η of 17*GPas* for the control specimens (Figure 2, right); a value that is 60% higher than the one computed for the aged fascicle specimens. The reduction pertains to the short-term relaxation time of fascicles, as the experimental curves (Figure 1C) restrain to a relaxation experiment of 200 s for each loading increment, a time-frame that is considerably shorter than the one required for a complete fascicle relaxation (*t* > 400*s*) in different studies (Machiraju et al., 2006; Davis and De Vita, 2012). Note that contrary to the substantial alteration of the time-dependent properties (see section Relaxing Healthy and Aging Fascicles), the fascicle's linear elastic attributes remain practically unaffected (Figure 2, right) (Gautieri et al., 2017). In particular, the elastic modulus is subject to a comparatively insignificant variation in the order of 5% (Figure 2, right). The rather negligible sensitivity of the elastic modulus to aging effects has been experimentally shown to apply, not only to the fascicle scale, but also to the inner scales of fibers and fibrils (Legerlotz et al., 2013; Li et al., 2013; Gautieri et al., 2017).

While the mean viscosity of control tendon fibers (Figure 3, left) well-compares to the short term viscosity of healthy tendon fascicles (Figure 3, left), the behavior of their aged counterparts differs to a large extend. More specifically, at the fiber scale, the viscosity decreases by approximately an order of magnitude (Figure 3, right), contrary to the 60% reduction in mean viscosity terms observed at the fascicle scale. The difference suggests that aging has a more predominant effect at the time-dependent properties of the lower tendon subunits. However, the observed viscosity changes are statistically significant both for tendon fascicles and fibers (see section Statistical significance of the Effective Viscosity Alterations Upon Aging).

The range of matrix viscosity values η_*m*_ reported in section Effective Viscosity Contributions of the Embedding Matrix constitute the first estimates –to the author's best knowledge- of the effective viscous contribution of the embedding matrix substance at the fiber scale that is based on experimental data. It needs to be noted that data-based estimates of the kind can be primarily obtained through the coupling of multiscale mechanical information (Karathanasopoulos et al., 2017, 2019; Karathanasopoulos and Ganghoffer, 2019a), as direct experimental testing is rather infeasible (Ault et al., 1992). The range of magnitudes computed for the effective embedding matrix viscosity η_*m*_ (Figure 4) suggests that the effective contribution of the matrix in the relaxation process at the fiber and fascicle scale is significant, both for the control and aged specimens. Analogous conclusions have been derived for the innermost tendon subunits of fibrils (Karathanasopoulos et al., 2019). What is more, for the case of aged tendon subunits, while the magnitude of the matrix viscosity is on average lower than the one obtained for the control specimens (Figure 4, left), their relative viscoelastic contribution is higher (Figure 4, right). The computed viscosity ratios (Figure 4, right) indicate that aging affects primarily the fibrillar components, rather than the embedding matrix substance.

We note that the current analysis has been restricted to available experimental studies on the effect of aging on the relaxation behavior of both lower and upper tendon subunits subunits (Li et al., 2013; Gautieri et al., 2017). The provided experimental information restrains to quasi-static relaxation experiments, which do not quantify the variance of the relaxation experiments for the specific strain magnitude or strain rate selected, factors that have been shown to play a role in the mechanical response of biological tissues (Zanetti et al., 2012; Natali et al., 2015). For such effects to be accounted for, further multiscale experimental data of the kind would be required, not only to decrease the reported overall experimental uncertainty (Figures 2–4), but also to provide experimental information that is up to now unavailable. In particular, separate short-time and long-time relaxation experiments could be carried out at different strain magnitudes and strain rates, to provide additional secondary information on the relaxation spectrum of each tendon subunit. Data of the kind could be thereafter used a basis for a more elaborate identification of the mechanical parameters at each tendon scale, as well as for the understanding of the functional role of the multiscale tendon architecture (Karathanasopoulos et al., 2019).

## Conclusions

Overall, the viscosity parameters computed in sections Relaxing Healthy and Aging Fascicles and Fiber Scale Aging Relaxation have provided primal, data-based quantitative estimates of the effect of aging on the time-dependent behavior of fascicles and fibers. It has been shown that the viscosity coefficients η_*fasc*_ and η_*f*_ are subject to statistically significant reductions as a result of the aging process (see section Statistical Significance of the Effective Viscosity Alterations Upon Aging). What is more, the effective viscous contribution of the embedding matrix substance η_*m*_ has been quantified, using a multi-scale mechanical analysis framework (see section Effective Viscosity Contributions of the Embedding Matrix). It has been shown that the matrix effective viscosity η_*m*_ is comparable to the one computed for the tendon's fibrillar components, while its viscoelastic contribution is higher for aged rather than for control, healthy tendon subunits. The results can be used as reference viscosity mechanical parameters, factors of primal importance for the understanding of the tendon's tissue mechanics, as well as for its regeneration (Sandri et al., 2016). We aspire that the identified viscosity parameters along with the elaborated methodology will serve as an engineering basis for the design of biocompatible restoration materials, as well as for the development of mechanically appropriate medical treatments for aged tendons (Legerlotz et al., 2013).

## Author Contributions

NK: conception, design, computations, and main editing. JG: analysis and interpretation of results.

### Conflict of Interest Statement

The authors declare that the research was conducted in the absence of any commercial or financial relationships that could be construed as a potential conflict of interest.
