# Supplementary material for: Investigating the Effect of Aging on the Viscosity of Tendon Fascicles and Fibers
Source: Front Bioeng Biotechnol. 2019 May 15;7:107. doi: 10.3389/fbioe.2019.00107 (PMC6529838; doi:10.3389/fbioe.2019.00107)
Supplement: Supplementary file 1 [file Data_Sheet_1.docx]

Appendix A

The relaxation curves for the control and aged fascicle and fiber specimens of Sections 3.1 and 3.2 are summarized in Fig. 5. The moduli evolution is depicted in a normalized form. The normalization to be carried out with respect to the elastic, time-independent stiffness. The errorbars reflect the variability of the curves within one standard deviation of their mean value.


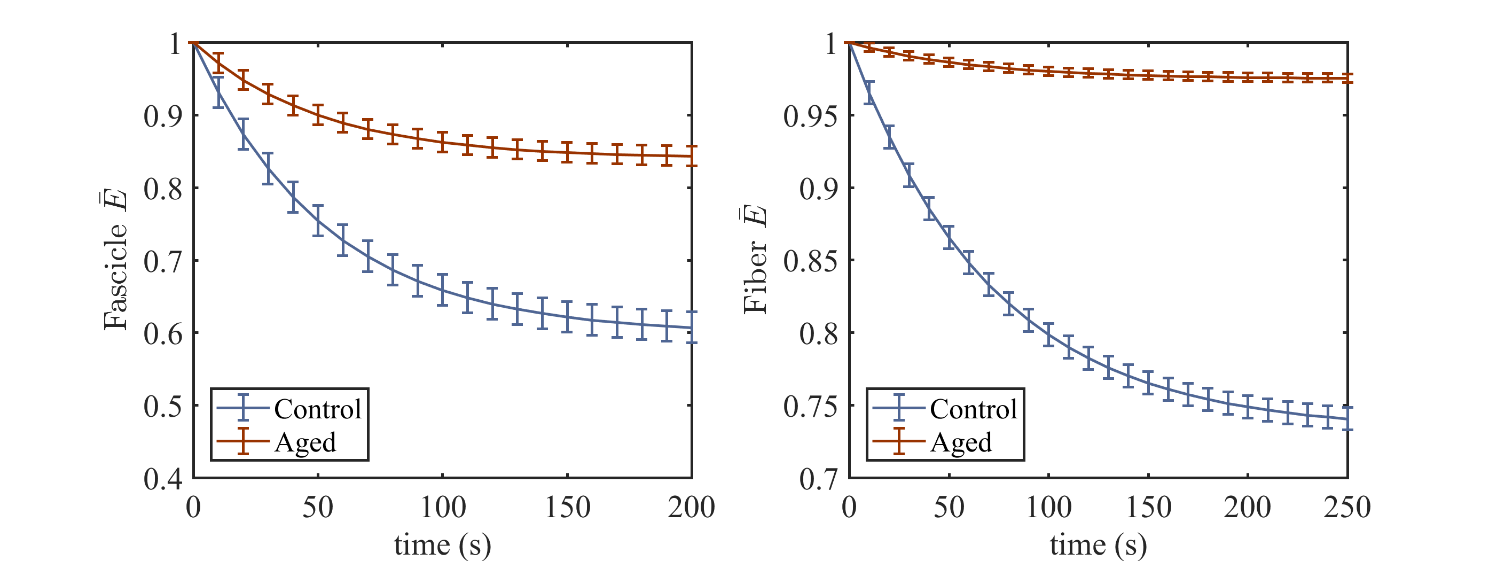


*Fig. 5: Fitted relaxation curves at the fascicle (left) and fiber (right) scale for control and aged specimens*
